# The effects of baking conditions on acrylamide content in shortcrust cookies with added freeze-dried aqueous rosemary extract

**DOI:** 10.1007/s13197-018-3349-x

**Published:** 2018-07-28

**Authors:** Karolina Miśkiewicz, Ewa Nebesny, Justyna Rosicka-Kaczmarek, Dorota Żyżelewicz, Grażyna Budryn

**Affiliations:** Faculty of Biotechnology and Food Sciences, Institute of Food Technology and Analysis, Lodz University of Technology, Stefanowskiego Street 4/10, 90-924 Lodz, Poland

**Keywords:** Acrylamide, DPPH radical scavenging capacity, Total phenolic content, Redox potential

## Abstract

The aim of the present study was to determine the effects of baking air humidity and dough supplementation with freeze-dried aqueous rosemary extract on acrylamide content in shortcrust cookies, as well as on their antioxidant properties and phenolic composition. Shortcrust cookies were baked at 170 °C in dry or humid (90%) air using 0.1, 0.2, or 0.5% of rosemary extract, and were compared to control samples without the extract. Acrylamide concentration in the obtained products ranged from 22.49 to 28.38 µg kg^−1^. Furthermore, cookies baked in humid air had less acrylamide (by 6% on average) than those baked in dry air, irrespective of extract content. On the other hand, a significant relationship was found between humidity conditions and total phenolic content in the final products. Shortcrust cookies containing 0.5% of rosemary extract and baked in humid air revealed the best antioxidant properties as their total polyphenol content was approx. Three times higher than that in control samples. Furthermore, shortcrust cookies with 0.5% of rosemary extract showed superior DPPH radical scavenging capacity.

## Introduction

While the thermal processing of carbohydrate-containing foods induces beneficial effects, such as the formation of desirable flavor, aroma, and color compounds, it may also generate some anti-nutritive substances, namely acrylamide and its derivatives (Rannou et al. 2016).

In April 2002, researchers from the University of Stockholm submitted a report to the UK Institute of Food Science and Technology concerning the formation of acrylamide in French fries, potato chips, and other food products, including bread. They presented the results of research conducted together with the Swedish National Food Administration, which drew attention to the high levels of this substance in thermally processed foods (Mustafa et al. 2008; Constantinou and Koutsidis 2016).

The thermal processing of food products gives rise to high molecular weight polymers and copolymers known as melanoidins, as well as low molecular weight compounds, such as furfural, acrolein, and acrylamide. It should be noted that acrylamide is formed in the course of Maillard reactions, together with compounds responsible for the flavor and aroma of browned products (Palermo et al. 2016; Friedman 2015).

In addition to the content and proportions of acrylamide precursors, i.e., asparagines and reducing sugars, the pH and water activity of the reaction medium (dough) are the main contributors to the formation of acrylamide in bread (Mustafa et al. 2008).

Acrylamide is potentially toxic to humans and its consumption may be linked to a number of diseases. In 1994, it was classified as “potentially carcinogenic to humans” by the International Agency for Research on Cancer (IARC). Acrylamide is neurotoxic and genotoxic (causing cell damage at a genetic level), adversely affects fertility, and induces cancers in animals (Kocadağlı et al. 2012).

Therefore, acrylamide content in foods must be controlled and reduced, for instance by decreasing the baking temperature, lowering pH conditions, as well as adding organic acids (mainly citric), calcium or magnesium ions, the enzyme asparaginase, and extracts with antioxidant properties (Salazar et al. 2012b, 2014).

Since chemical compounds exhibiting antioxidant properties are present in many herbs and spices (Rachwał and Nebesny 2012), one method of reducing acrylamide content in food products is the addition of plant extracts, and especially those from rosemary, oregano, sage, and thyme. The phenolic compounds they contain are mainly diterpenes, phenolic acids, and flavonoids. Specifically, the components responsible for the antioxidant properties of rosemary are carnosol, epirosmanol, and methyl carnosine. Moreover, sixteen other compounds, including flavonones, steroid diterpenes, and triterpenes have been isolated from rosemary. According to O’Grady et al. (2006), the activity of rosemary extracts is associated with carnosic acid and carnosol, which exhibit greater antioxidant activity than β-tocopherol, a natural antioxidant.

The literature contains several reports on the use of natural antioxidants to suppress acrylamide formation in food. Schamberger and Labuz (2007) found that epicatechin and epigallocatechin gallate lowered the amount of Maillard reaction products during UHT milk sterilization. Zhang and Zhang (2008), who investigated acrylamide formation and elimination in an aqueous asparagine–glucose system with the addition of antioxidants, also obtained a significant decline in the concentration of this carcinogen. Zhang et al. (2007) lowered the content of acrylamide in potato chips and French fries by supplementation with a bamboo extract. In turn, Hedegaard et al. (2008) demonstrated a significant effect of aqueous rosemary extract in bread dough on acrylamide content in the final product (in this case ground rosemary leaves were combined with water in the ratio 1:20 w/v and extracted in the dark at room temperature under stirring for 10 min).

Given the above, the aim of the present study was to determine the effects of baking air humidity and dough supplementation with freeze-dried aqueous rosemary extract on the content of acrylamide in shortcrust cookies, as well as on their antioxidant properties and phenolic composition.

## Materials and methods

### Raw materials for the preparation of freeze-dried aqueous rosemary extract

Rosemary (*Rosmarinus officinalis* L.) originating from Morocco was supplied by Ziołopex (Stawiszyn, Poland). Prior to extraction, dry rosemary leaves were ground in a mill to a particle size of 0.1–0.5 mm. The ground material was then subjected to extraction with distilled water in a 1:10 ratio. Extraction was carried out at elevated pressure (202 kPa) in a First PS-5682 pressure vessel (Vienna, Austria) at 110 °C for 10 min. Then, the obtained extracts were centrifuged at 4000 rpm for 15 min, filtered under reduced pressure using a KNF Neuberger N 035.3 AT.18 vacuum pump (Germany), and subjected to freeze-drying in a Christ DELTA 1-24LSC freeze dryer (Osterode AM Harz, Germany). The freeze-dried preparation of rosemary used in this study was characterized by a total phenolic content of 37.21 mg 100 g^−1^ solid substance (s.s.) and the ability to scavenge DPPH at a level of 0.10 mg mg^−1^ DPPH.

### Raw materials for the preparation of shortcrust cookies

The following ingredients were used to obtain shortcrust cookies: wheat flour type 500 (Diamant Int. Poland), “Kasia” margarine (Unilever), yolks from fresh Class A eggs, white sugar (Nordzucker Poland), and baking powder containing disodium pyrophosphate and sodium bicarbonate (Delecta).

### Preparation of shortcrust cookies

Shortcrust cookies were prepared according to the following recipe: flour (149 g), margarine (99 g), sugar (99 g), egg yolks (49 g), and baking powder (2 g). Freeze-dried aqueous rosemary extract was added in an amount of 0.1, 0.2, and 0.5% at the expense of flour. The cookies were baked in an Ariston C 3 VP6 electric oven at 170 °C for 10 min in dry or humid (90%) air.

### Analyses of dough and shortcrust cookies

#### Solid substance content

Solid substance content in both dough and baked shortcrust cookies was measured by the gravimetric method at 102 °C until constant weight (a difference of less than 0.004 g after 30 min of drying), according to the standard PN-91/A-74010:^1991^.

#### Water activity

Water activity in both dough and baked shortcrust cookies was measured using a Hygropalm AW-1 apparatus (Rotronic AG, Bassersdorf, Switzerland). Dough or crumbled cookies (approx. 2 g) were placed in a WP-40 vial, sealed, and left at 23 °C for approx. 30 min. Then, the opened vial was put on the apparatus plate and plugged with an AW-DIO probe.

#### pH determination

The pH of the dough and shortcrust cookies was measured using a Schott CG 843 pH-meter with a BlueLine 11 electrode (Schott Geräte GmbH, Mainz, Germany).

#### Texture analysis

The texture of the dough and baked shortcrust cookies was measured using a TA.XT plus Stable Micro System Texture Analyzer (Godalming, UK) according to the AACCI Method 74-09.01 (2011).

Dough firmness was determined using an A/DP rig. A 6 mm cylinder probe penetrated the sample to a depth of 20 mm with a speed of 3 mm s^−1^.

Dough stickiness was measured with the use of an A/DSC rig equipped with a lapped finish cylinder probe with a piston driven by a screw. A dough sample was placed in the cylinder. The cylinder was then closed with a lid with extrusion holes, through which the dough was extruded to a length of 1 mm. Subsequently, a Perspex cylinder probe with a 25 mm diameter penetrated the sample to a depth of 5 mm with a speed of 0.5 mm s^−1^. The contact time between the probe and the sample was 0.1 s.

The hardness of baked shortcrust cookies was measured using a HDP/3 PB rig. In this test, a cookie was placed on the lower supporting blades spaced 40 mm apart. Then, the cookie was broken by the upper blade, which penetrated the sample to a depth of 3 mm with a speed of 1 mm s^−1^ and a pressing force of 5 g.

#### Color measurement in the CIE L*a*b* system

The color of the baked shortcrust cookies was measured using an automatic CR-400 Konica Minolta colorimeter with Spectra Magic NX 1.3 software (Osaka, Japan). The color parameters were L*—brightness (from 0—black to 100—white), a*—(from − 50—green to 50—red), and b*—(from − 50—blue to 50—yellow).

#### Sensory analysis

Sensory analysis was carried out according to the standard PN-A-74252:^1998^ specifying sensory methods for pastry analysis. The finished products were scored on such quality factors as batch uniformity, appearance, structure and texture, flavor, and scent. Analysis was performed in a laboratory specially equipped for that purpose by a panel consisting of 10 persons with previously proven high sensory sensitivity, who had been trained in evaluating the studied attributes. The overall quality of the product was calculated based on the scores for individual attributes rated on a 5-level scale with 1 denoting extremely poor quality and 5 corresponding to typical, characteristic, and highly acceptable attributes.

## Antioxidant properties of shortcrust cookies

### Redox potential

Redox potential of 25 mL of suspension (2 g of ground shortcrust cookies per 100 g of water) was tested using a Shott CG 843 voltmeter (Schott, Mainz, Germany) equipped with a platinum BlueLine 31RX electrode according to Budryn et al. (2013). Calibration was performed against a redox standard solution (Reagecon Diagnostics Ltd., Shannon, Ireland). Redox potential was monitored at 25 °C for at least 5 min, until an arbitrarily stable result was achieved, defined as a change of less than 2 mV over 3 min.

### Preparation of phenolic extracts from shortcrust cookies

Extracts were prepared by adding 91 mL of extraction mixture (i.e., 80 mL of methanol, 10 mL of doubly distilled water, and 1 mL of concentrated HCl) to a 20 g sample of ground cookies. Extraction was carried out in a shaker at 30 °C for 120 min. Then, the sample was centrifuged for 10 min (6000 rpm, 4 °C), filtered through a Büchner funnel, and defatted twice with 10 mL of hexane. The upper hexane layer was decanted and the extract was concentrated to a volume of 25 mL by rotary evaporation at 40 °C (Büchi V-855, R-210/215 with a V-700 vacuum pump).

### Determination of total phenolic content

Total phenolic content was determined spectrophotometrically using Folin–Ciocalteu reagent according to a modified method by Żyżelewicz et al. (2014).

#### Sample preparation

First, 5 g of extract from shortcrust cookies (weighed with an accuracy to 0.0001 g) was placed in a 50 mL volumetric flask, made up to the mark with 80% methanol, and filtered. Then, 2 mL of the filtrate was withdrawn to a 50 mL volumetric flask. Subsequently, 4.2 mL of distilled water and 0.5 mL of Folin–Ciocalteu reagent were added, and the mixture was stirred for 1 min. Finally, 1 mL of 20% sodium carbonate solution was added and the mixture was made up to the mark with distilled water.

#### Reference sample preparation

In a 50 mL volumetric flask, 0.5 mL of Folin–Ciocalteu reagent was mixed with 0.5 mL of 80% methanol solution, 5 mL of 20% sodium carbonate solution, and made up to the mark with distilled water.

The absorbance of the prepared samples, left for 2 h in the dark, was measured at 760 nm against the reference sample using a Hitachi UV/VIS U-2800 A spectrophotometer (Tokyo, Japan). Total phenolic content was determined on the basis of a calibration curve prepared for gallic acid.

### Spectrophotometric determination of DPPH radical scavenging capacity

The scavenging effects of the studied extracts were determined for 2,2-diphenyl-1-picrylhydrazyl (DPPH) radical according to a modified method of Scherer and Godoy (2009).

#### Sample preparation

Different concentrations of the extract diluted with distilled water, i.e., 1:2, 1:5, 1:10, and 1:20, were prepared in 50 mL volumetric flasks. At the same time, 5 mg of DPPH radical was transferred to a 100 mL volumetric flask and made up with methanol. Subsequently, 7.8 mL of this solution was placed in test tubes, and 0.2 mL of extracts at different concentrations was added to each. Absorbance was measured at 517 nm after 30 min incubation using a Hitachi UV/VIS U-2800 A spectrophotometer (Tokyo, Japan). Measurements were made against a reference sample containing 7.8 mL of methanol and 0.2 mL of the extract at an appropriate concentration.

#### Control sample preparation

7.8 mL of DPPH solution was placed in a test tube and 0.2 mL of distilled water was added. Measurement was done after 30 min incubation, at 517 nm, using a Hitachi UV/VIS U-2800 A spectrophotometer (Tokyo, Japan).

Calculations were made based on the equation below:

$$ {\text{Scavenging activity}}\,\left( \% \right) = \left( {\frac{{{\text{A}}_{517} \,{\text{of}}\,{\text{control}} - {\text{A}}_{517} \,{\text{of}}\,{\text{sample}}}}{{{\text{A}}_{517} \,{\text{of}}\,{\text{control}}}}} \right) \times 100 $$

where A_517_ of control—absorbance of the control sample, A_517_ of sample—absorbance of the sample.

The radical scavenging activity of a given solution is proportional to its concentration. EC_50_ is defined as the amount of solid substance in the extract causing a 50% reduction of the initial DPPH content and calculated from the equation describing the relationship between the residual content of DPPH and the concentration of the added extract.

### Qualitative and quantitative analysis of phenolic compounds by HPLC

Qualitative and quantitative analysis of phenolic compounds in the tested extracts from shortcrust cookies was performed by HPLC according to the procedure described by Oracz et al. (2015) with some modifications.

#### Sample preparation

Extracts were prepared by adding 91 mL of extraction mixture (i.e., 80 mL of methanol, 10 mL of doubly distilled water, and 1 mL of concentrated HCl) to a 20 g sample of ground cookies. Extraction was carried out in a shaker at 30 °C for 120 min. Then, the sample was centrifuged for 10 min (6000 rpm, 4 °C), filtered through a Büchner funnel, and defatted twice with 10 mL of hexane. The upper hexane layer was decanted and the extract was passed through an SPE (C18) column conditioned by washing twice with 4 mL of HPLC-grade methanol and once with 4 mL of distilled water. After passing the defatted extract through the columns, they were washed with 3 mL of methanol to elute the remaining polyphenols. The purified sample was concentrated to dryness by rotary evaporation at 40 °C (Büchi V-855, R-210/215 with a V-700 vacuum pump). The dry residue was then dissolved in 5 mL of HPLC-grade methanol, 1 mL of solution was withdrawn, and 5 mL of HPLC-grade methanol was again added to it. This solution was filtered through a nylon membrane filter with a 0.2 μm pore diameter, poured into a 2 mL autosampler vial made of dark glass, and subjected to chromatographic analysis.

#### Chromatographic analysis

Chromatographic separation was performed using a HPLC + Ultimata 3000 chromatograph from Dionex, coupled with a UV–Vis detector and a Tosoh Bioscience TSKgel ODS-100Z column (150 mm × 4.6 mm, 5.0 µm) thermostated at 40 °C, with 5 µL injection volume. A gradient elution mode was used: 1% formic acid in water (phase A)—1% formic acid in water and acetonitrile (20:80 v/v) (phase B), at a flow rate of 1.0 mL min^−1^. Separation was performed with the following gradient program: 0 min, 5% B; 15 min, 15% B; 30 min, 20% B; 35 min, 25% B; 60 min, 80% B; 62 min, 100% B; 65 min, 5% B; 70 min, 5% B. Standard solutions were used for the identification of phenolic compounds, and determinations were performed by measuring absorbance at two wavelengths: λ = 280 nm for carnosic and vanillin acids and λ = 320 nm for caffeic and rosmarinic acids.

### Identification of phenolic compounds by UHPLC/DAD–ESI–MS/MS

The identification of phenolic compounds in rosemary extract and in shortcrust cookies supplemented with it was performed according to Budryn et al. (2014) with some modifications, using a TurboFlow UHPLC/DAD–ESI–MS/MS chromatograph equipped with a Thermo Scientific Q-Exactive tandem mass spectrometer (Hudson, NH, USA). The UHPLC system consisted of a TurboFlow P 50 × 0.5 mm cyclone purification column and an Accucore C18 100 × 3 mm, 2.6 μm analytical column, both from Thermo Scientific (Hudson, NH, USA), equipped with a loading and analytical pump, respectively. After injecting 10 µL of the sample onto the TurboFlow column, chromatographic analysis was performed at 30 °C. A gradient elution mode was used: 1% formic acid in water (phase A)—1% formic acid in water and acetonitrile (20:80 v/v) (phase B).

Identification of phenolic compounds was carried out using a Q-Exactive hybrid quadrupole-Orbitrap mass spectrometer with Thermo Xcalibur 2.2 and Qexactive Tune 2.1 software. The data necessary to identify phenolic compounds were collected in full scan MS and target MS2 modes, with mass spectra being recorded in the negative ionization mode in the range from *m/z* 100 to 1500. The capillary temperature was 320 °C. Nitrogen served as both nebulizer and collision gas. Collision energy was 35 eV and ionization voltage equaled 3.0 kV.

### Acrylamide content

Acrylamide was quantified in baked cookies by GC–MS/MS after derivatization, using GC–MS/MS parameters according to Mojska (2008) and Soares and Fernandes (2009).

#### Sample preparation

A 2 g sample of ground shortcrust cookies weighed with an accuracy of 0.0001 g was placed in a 50 mL centrifuge tube, and 100 µL of 100 µg mL^−1^ deuterated acrylamide standard solution and 20 mL of doubly distilled water were added. The centrifuge tube was placed in a shaking water bath at 60 °C for 30 min. After cooling to room temperature, the extract was centrifuged (20 min, 6000 rpm, 4 °C). The precipitate was discarded and the supernatant was defatted three times with decreasing volumes of hexane: 20, 15, and 10 mL, in consecutive steps. The resulting mixture was shaken for 1 min and then centrifuged for 10 min (6000 rpm, 10 °C). The upper hexane layer was discarded and 0.3 mL of Carrez I and II reagents was added to the lower aqueous layer, which was then slightly shaken and centrifuged for 10 min (6000 rpm, 10 °C). The obtained extract was brominated overnight. For this purpose, 2.5 g of KBr, 0.1 mL of HBr (pH 1–3), and 2.5 mL of bromine water were added. Bromination was performed in an ice bath (~ 0 °C) without light access.

Excess bromine was degraded with a few drops of 1 M sodium thiosulfate. The disappearance of the yellow color determined the end of the reaction. Then, 4 g of NaCl was added, mixed until dissolution, and extraction with ethyl acetate was performed twice: 4 mL of ethyl acetate was added to the solution, which was then shaken for 5 min. After phase separation, the ethyl acetate layer was collected, dried over anhydrous sodium sulfate (~ 4 g), and centrifuged for 10 min (6000 rpm, 4 °C). The organic phase of the extract was evaporated to dryness at 40 °C and the solids were dissolved in 1 mL of ethyl acetate. Samples were then transferred to 2 mL autosampler vials made of dark glass.

#### GC–MS/MS analysis

The 2,3-dibromo derivative of acrylamide was quantified by GC using a Varian 450-GC gas chromatograph equipped with an ion trap mass detector (Varian 220-MS) and a split/splitless injector. Analytical separation was performed on a Varian Factor Four VF-5 ms capillary column (0.25 µm film thickness, 30 m 0.25 mm i.d.) (Varian 220-MS). Samples (1 µL) were analyzed at an ionization energy of 70 eV. In the first step, precursor ions with *m*/*z* 152 and 155 were obtained from 2,3-dibromo derivatives of acrylamide and deuterated acrylamide, respectively. Their collisions gave rise to daughter ions with *m*/*z* 135 (from *m*/*z* 152 ions) and *m*/*z* 137 (from *m*/*z* 155 ions). Acrylamide concentration in the tested samples was calculated from the ratio of areas under peaks corresponding to ions with *m*/*z* 135 and 137.

The temperature increased from 65 to 240 °C at a rate of 15 °C min^−1^. The injector temperature was 250 °C, the carrier gas was helium, and the flow rate was 40 mL s^−1^. The MS parameters were as follows: ionization energy 70 eV, ion source temperature 180 °C, transfer line temperature 250 °C.

#### Method performance characteristics

Quantification was performed by the internal standard method. A calibration curve was constructed by plotting the ratio Aaa/Ais against Caa/Cis, where Aaa is the area of unlabeled acrylamide as mass trace *m*/*z* 135, Ais is the area of deuterium-labeled acrylamide as mass trace *m*/*z* 137, and Caa/Cis is the concentration ratio of acrylamide and 2,3,3-*d*3-acrylamide. The calibration curve was prepared in the range of 2.5–500 µg L^−1^.

The limits of detection and quantification (LOD and LOQ) of the method were calculated using the calibration curve parameters. In this case, the LOD was 2.5 μg kg^−1^ and the LOQ was set at 5 μg kg^−1^.

Recoveries were determined by adding 50 µg L^−1^ of acrylamide standard solution to a sample of shortcrust cookies. Average recoveries ranged from 73 to 89%.

### Statistical analysis

Each variant of shortcrust cookies was baked in three batches. All analyses were carried out in triplicate with the results being given as means with standard deviations. The significance of differences was determined using Tukey’s *t* test. Results were accepted as statistically significant at *p *< 0.05. Statistical data are shown in tables and figures; results which are statistically significantly different are labeled with different letters.

## Results and discussion

### Physicochemical analysis of dough and shortcrust cookies

Dough supplemented with freeze-dried aqueous rosemary extract contained from 14.25 to 15.07% of water for 0.1 and 0.2% extract concentrations, respectively (Table 1a). It was found that water content rose with the dose of rosemary extract in the dough. Baking decreased water content in the finished products down to 3.95%, which translates into an approx. 73% reduction as compared to the dough (Table 1b, c) both in the case of dry and humid (90%) air conditions. Shortcrust cookies containing rosemary extract, whether baked in dry or humid air, contained less water than those without the extract (Table 1b, c). This may be due to the fact that the water binding capacity of the dough ingredients increased in the presence of the extract, lowering water evaporation in the process of baking.

**Table 1:** Physicochemical properties of the dough (a) and shortcrust cookies baked under dry (b) and humid (c) air conditions at 170 °C depending on the concentration of freeze-dried aqueous rosemary extract

Concentration of the applied freeze-dried aqueous rosemary extract (%)	Water content (%)	a_w_	pH	Firmness (g)	Stickiness (g s)
(a)					
Control sample	14.74^a^ ± 0.15	0.718^a^ ± 0.003	6.75^a^ ± 0.01	127.90^a^ ± 7.81	33.99^a^ ± 2.75
0.1	14.25^b^ ± 0.11	0.694^b^ ± 0.001	6.82^b^ ± 0.01	115.55^b^ ± 2.33	40.77^b^ ± 1.12
0.2	15.07^c^ ± 0.12	0.714^c^ ± 0.001	6.78^c^ ± 0.01	151.67^c^ ± 12.05	39.79^c^ ± 1.10
0.5	14.29^d^ ± 0.02	0.765^d^ ± 0.005	6.73^d^ ± 0.03	158.03^c^ ± 8.34	38.15^d^ ± 1.10

Concentration of the applied freeze-dried aqueous rosemary extract (%)	Water content (%)	a_w_	pH	Hardness (kg)	Color			Sensory assessment	
					L*	a*	b*	Total number of points	Quality level
(b)									
Control sample	3.83^a^ ± 0.06	0.252^a^ ± 0.002	6.77^a^ ± 0.09	0.801^a^ ± 0.037	79.59^a^ ± 0.06	1.02^a^ ± 0.02	30.19^a^ ± 0.09	20.00	I
0.1	3.32^b^ ± 0.05	0.230^b^ ± 0.004	7.20^b^ ± 0.01	0.961^b^ ± 0.142	77.76^b^ ± 0.02	0.83^b^ ± 0.05	28.82^b^ ± 0.07	18.00	I
0.2	3.71^c^ ± 0.02	0.249^c^ ± 0.004	7.13^c^ ± 0.02	0.966^b^ ± 0.079	76.09^c^ ± 0.07	1.75^c^ ± 0.09	31.23^c^ ± 0.09	18.00	I
0.5	4.84^d^ ± 0.01	0.265^d^ ± 0.004	7.12^c^ ± 0.05	0.647^c^ ± 0.088	73.49^d^ ± 0.08	2.66^d^ ± 0.03	31.00^d^ ± 0.07	16.00	II
(c)									
Control sample	4.15^a^ ± 0.02	0.336^a^ ± 0.005	6.97^a^ ± 0.03	0.589^a^ ± 0.049	78.56^a^ ± 0.08	2.94^a^ ± 0.05	32.51^a^ ± 0.06	19.00	I
0.1	3.82^b^ ± 0.03	0.268^b^ ± 0.000	6.93^a^ ± 0.11	0.981^b^ ± 0.023	76.55^b^ ± 0.08	2.79^b^ ± 0.08	31.51^b^ ± 0.09	18.00	I
0.2	4.06^a^ ± 0.08	0.291^c^ ± 0.009	7.08^b^ ± 0.03	0.952^c^ ± 0.20	75.08^c^ ± 0.02	3.13^c^ ± 0.09	32.81^c^ ± 0.07	17.00	II
0.5	3.96^c^ ± 0.01	0.350^d^ ± 0.001	7.02^c^ ± 0.01	0.870^d^ ± 0.018	71.62^d^ ± 0.06	5.27^d^ ± 0.34	33.35^d^ ± 0.08	15.00	II

Data are presented as mean ± SD (n = 3)

Control sample-shortcrust cookies without rosemary extract

Within each row values marked with superscript small letters differ significantly (*p* < 0.05)—compare changes between different concentrations of rosemary extract

The water activity of the dough ranged from 0.694 to 0.765 for samples supplemented with 0.1 and 0.5% of rosemary extract, respectively (Table 1a). Baking under both dry and humid air conditions reduced this parameter by 65.5 and 57%, respectively, as compared to the dough (Table 1b, c). Furthermore, the water activity of both the dough and finished products increased with rosemary extract concentration. The pH of the dough and shortcrust cookies was similar at 6.77 (Table 1a) and 7.03 (Table 1b, c), respectively.

Analysis of dough stickiness showed that the addition of rosemary extract with antioxidant properties caused an increase in this parameter by approx. 16.4% as compared to dough without the extract (Table 1a). Stickiness was also found to decrease with rosemary extract concentration. Furthermore, as can be seen from Table 1a, dough firmness was directly proportional to the concentration of rosemary extract in the recipe. According to Selinheimo et al. (2008), the differences in firmness and stickiness may be caused by covalent bonding reactions between wheat flour proteins and the polyphenols introduced with the extract.

Furthermore, an increase in baking air humidity from dry to 90% made the products supplemented with 0.1 and 0.5% of the extract harder by approx. 2.08 and 34.5%, respectively (Table 1b, c). In the case of cookies without rosemary extract and with 0.2% of the extract, higher air humidity decreased hardness by 26.5 and 1.45%, respectively.

The color of shortcrust cookies containing different concentrations of rosemary extract was also examined. This color is formed as a result of Maillard reactions, which are non-enzymatic browning reactions dependent on the content of reducing sugars in the product and on baking temperature (Pedreschi et al. 2006; Friedman 2015). The following parameters were determined in the baked products: color brightness (L*), the degree of redness (a*), and the degree of yellowness (b*). As can be seen from Table 1b, c, L* decreased with increasing concentration of rosemary extract, leading to cookies of a darker color. On the other hand, the chromatic components a* and b* tended to increase with extract concentration, making the baked products redder and yellower.

The studied shortcrust cookies were also rated in terms of their sensory attributes. The highest scores (20 and 19 points) were obtained by cookies without the extract, baked in dry (Table 1b) and humid air (Table 1c), respectively. Cookies supplemented with 0.5% of rosemary extract, baked under these humidity conditions, exhibited the greatest change in color and taste with respect to the reference sample, scoring 16 and 15 points, respectively. Nevertheless, although they were classified as 2nd quality, the evaluators’ low gustatory ratings did not disqualify the products with rosemary extract.

### Acrylamide content in shortcrust cookies

In view of the fact that a series of reactions leading to acrylamide formation takes place in the process of dough baking (Gokmen et al. 2007), the present study analyzed the effects of rosemary extract concentration and baking air humidity on the content of this toxic compound in the finished product.

**Fig. 1:**
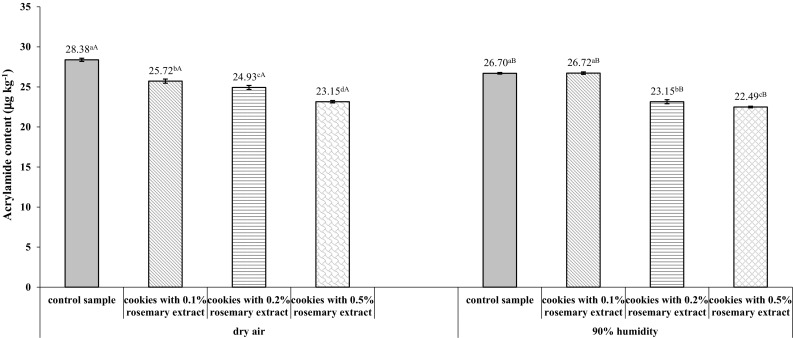
Acrylamide content in shortcrust cookies made from dough supplemented with different concentrations of freeze-dried aqueous rosemary extract and baked in dry or humid (90%) air. Control sample-shortcrust cookies without rosemary extract. Error bars represent standard deviations (± SD). Values are given as means from three replicates. Within each row values marked with superscript small letters differ significantly (*p *< 0.05)—compare changes between different concentrations of rosemary extract under the same air humidity conditions. Within each row values marked with superscript capital letters differ significantly (*p *< 0.05)—compare changes between the same concentrations of rosemary extract under different air humidity conditions

In the obtained cookies, acrylamide concentration ranged from 28.38 to 23.15 μg kg^−1^ for dry baking air and from 26.70 to 22.49 μg kg^−1^ for humid baking air (Fig. 1). The addition of rosemary extract to the dough resulted in reduced acrylamide formation in a concentration-dependent manner. The greatest decrease was observed in cookies containing 0.5% of the extract (by 18.4 and 15.8% for dry and humid air conditions, respectively) as compared to products without the extract. The smallest reduction of acrylamide content was found in the presence of 0.1% of rosemary extract for both dry and humid baking air (9.4% and no significant change, respectively, see Fig. 1). These results are consistent with the complex mechanism of acrylamide formation and confirm that the presence of an antioxidant in the dough decreases the amount of this compound in shortcrust cookies.

Rosemary, known for its antioxidant properties, is used as an effective inhibitor of acrylamide formation in food. The literature describes reduced acrylamide concentrations in potato chips fried with both corn oil and olive oil with rosemary extract (Becalski et al. 2003). This is in agreement with a study by Hedegaard et al. (2007), who reported that acrylamide content declined by 62, 67, and 57% in bread made with the addition of aqueous rosemary extract, oil, and dried leaves, respectively, as compared to no additives. The lower degree of acrylamide content reduction in the analyzed shortcrust cookies (ranging from 15.8 to 18.4%) as compared to the much higher values obtained by Hedegaard et al. (2008) for bread may be explained by differences in the ingredients (shortcrust cookie dough has a high content of fat and sucrose, which are precursors to acrylamide). Another factor affecting the amount of this compound is temperature, especially within the product. According to the literature data, one of the factors necessary for acrylamide formation is a temperature above 120 °C (Palermo et al. 2016; Friedman 2015). The temperature inside bread does not exceed 100 °C during baking (at an air temperature of 220 °C), whereas that inside shortcrust cookies reaches the baking process temperature, i.e., 170 °C. It is also known that phenolic compounds are unstable at elevated temperatures (Alonso et al. 2002). These considerations may account for an almost twofold smaller reduction of acrylamide content in cookies in comparison to the literature data presented for bread (Hedegaard et al. 2008).

Research shows a strong correlation between the darkening of heat-treated products and their acrylamide content, mainly in food products not supplemented with antioxidants (Mustafa et al. 2005). Indeed, Salazar et al. (2012a) demonstrated that the supplementation of olive oil with pepper-derived antioxidants reduced acrylamide content in fried products despite their darker color. A similar tendency was observed in this study: decreased acrylamide levels in shortcrust cookies were found to coincide with increasing rosemary extract content and with decreasing color brightness.

### Antioxidant properties of shortcrust cookies

**Fig. 2:**
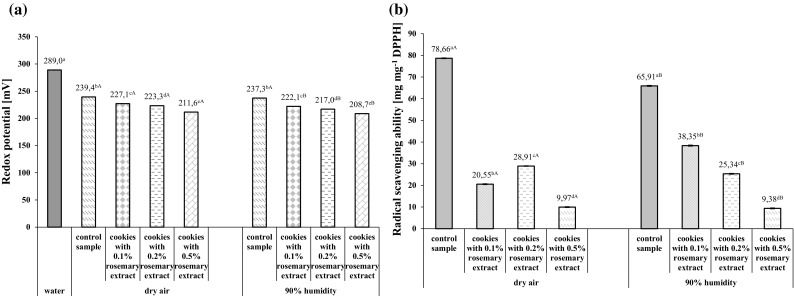
Changes in redox potential (**a**) and DPPH radical scavenging ability (**b**) in shortcrust cookies depending on the concentration of freeze-dried aqueous rosemary extract and baking air humidity. Control sample-shortcrust cookies without rosemary extract. Error bars represent standard deviations (± SD). Values are given as means from three replicates. Within each row values marked with superscript small letters differ significantly (*p *< 0.05)—compare changes between different concentrations of rosemary extract under the same air humidity conditions. Within each row values marked with superscript capital letters differ significantly (*p *< 0.05)—compare changes between the same concentrations of rosemary extract under different air humidity conditions

Redox potential is a measure of the ability to enter into chemical reactions resulting in oxidation or reduction processes. Since shortcrust cookies with freeze-dried rosemary extract, baked both in dry and humid air, revealed a significantly lower redox potential than water (Fig. 2a), they may be claimed to possess health benefits due to their greater reduction capacity. Furthermore, humid air conditions were found to enhance the antioxidant activity of shortcrust cookies containing rosemary extract by approx. 7% as compared to dry air. In addition, the antioxidant properties of the studied cookies increased in proportion to extract concentration in the dough regardless of baking air humidity (Fig. 2a).

**Fig. 3:**
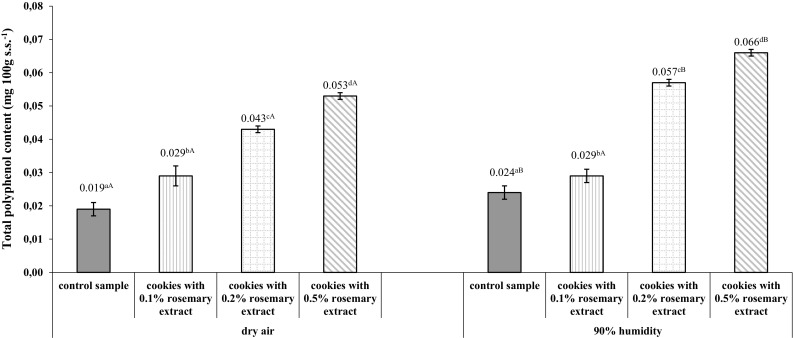
Changes in the total phenolic content of shortcrust cookies depending on the concentration of freeze-dried aqueous rosemary extract and baking air humidity. Control sample-shortcrust cookies without rosemary extract. Error bars represent standard deviations (± SD). Values are given as means from three replicates. Within each row values marked with superscript small letters differ significantly (*p *< 0.05)—compare changes between different concentrations of rosemary extract under the same air humidity conditions. Within each row values marked with superscript capital letters differ significantly (*p *< 0.05)—compare changes between the same concentrations of rosemary extract under different air humidity conditions

The total phenolic content of the studied shortcrust cookies ranged from 0.019 mg 100 g^−1^ s.s. for those without rosemary extract and baked in dry air to 0.066 mg 100 g^−1^ s.s. for those supplemented with 0.5% of the extract and baked in humid air (Fig. 3). As can be seen from Fig. 3, cookies baked in humid air contained on average 27% more polyphenols than those baked in dry air, regardless of the amount of extract added. Moreover, there was a strong positive correlation (correlation coefficient r = 0.99) between the antioxidant activity of extract and phenolic content for both dry and humid air conditions.

The analyzed shortcrust cookies were characterized by DPPH radical scavenging capacity ranging from 9.38 mg mg^−1^ DPPH for those containing 0.5% of freeze-dried rosemary extract and baked in humid air to 78.66 mg mg^−1^ DPPH for those without the extract and baked in dry air (Fig. 2b). The DPPH scavenging ability of the cookies was strongly correlated with their total phenolic content. It should be noted that this ability was greater in products baked in humid versus dry air, as confirmed by correlation coefficients (r = 0.86 and r = 0.77, respectively).

The antioxidant activity of shortcrust cookies with rosemary extract was shown to be negatively associated with acrylamide concentration. Correlation of total phenolic content and DPPH radical scavenging ability with acrylamide concentration was determined. It was found that an increase in the amount of the extract led to a higher total phenolic content with a simultaneous decrease in acrylamide concentration in the products, regardless of baking air humidity, as evidenced by the high correlation coefficients (r = − 0.96 for dry air and r = − 0.98 for humid air). Furthermore, it was also observed that the more rosemary extract was added to the dough, the higher the DPPH radical scavenging ability of the cookies and the greater the reduction of acrylamide concentration. This tendency was observed for products baked both in dry and humid air (r = 0.93 and r = 0.86, respectively).

### Phenolic composition of freeze-dried aqueous rosemary extract and shortcrust cookies

The phenolic composition of freeze-dried rosemary extract and shortcrust cookies made from dough supplemented with different concentrations of that extract was investigated by HPLC. The identified polyphenolic compounds were rosmarinic, carnosic, caffeic, and vanillic acids (Table 2). In rosemary extract, the dominant compounds were rosmarinic acid (7602.76 mg 100 g^−1^ s.s.) and carnosic acid (782.55 mg 100 g^−1^ s.s.), which are known to be predominantly responsible for the antioxidant properties of the rosemary plant (Kontogianni et al. 2013). Moreover, the extract contained 475.63 mg 100 g^−1^ s.s. of caffeic acid and 291.84 mg 100 g^−1^ s.s. of vanillic acid.

**Table 2:** Polyphenolic composition of shortcrust cookies supplemented with different concentrations of freeze-dried aqueous rosemary extract and baked under different air humidity conditions at 170 °C

Extract concentration in shortcrust cookies (%)	(mg 100 g^−1^ s.s.)							
	Dry air				Humid air (90%)			
	Rosmarinic acid	Carnosic acid	Caffeic acid	Vanillic acid	Rosmarinic acid	Carnosic acid	Caffeic acid	Vanillic acid
Control sample	nd	nd	0.125^aA^ ± 0.005	nd	nd	nd	0.169^aB^ ± 0.004	nd
0.1	0.166^aA^ ± 0.010	nd	0.236^bA^ ± 0.005	0.604^aA^ ± 0.010	0.142^aB^ ± 0.005	nd	0.196^bB^ ± 0.006	0.967^aB^ ± 0.003
0.2	0.316^bA^ ± 0.009	nd	0.237^bA^ ± 0.007	1.249^bA^ ± 0.011	0.529^bB^ ± 0.006	0.128^a^ ± 0.007	0.343^cB^ ± 0.008	1.389^bB^ ± 0.008
0.5	1.919^cA^ ± 0.007	0.288^A^ ± 0.006	0.414^cA^ ± 0.009	1.383^cA^ ± 0.010	1.525^cB^ ± 0.006	0.130^aB^ ± 0.004	0.374 ^dB^ ± 0.008	3.294^cB^ ± 0.011

Data are presented as mean ± SD (n = 3)

Control sample-shortcrust cookies without rosemary extract

Within each row values marked with superscript small letters differ significantly (*p* < 0.05)—compare changes between different concentrations of rosemary extract under the same air humidity conditions

Within each row values marked with superscript capital letters differ significantly (*p* < 0.05)—compare changes between the same concentrations of rosemary extract under different air humidity conditions

The main phenolic compounds in baked shortcrust cookies were vanillic and rosmarinic acids (Table 2). Their concentration was higher in cookies baked in humid air as compared to dry air. As can be seen from Table 2, caffeic acid was present both in cookies with and without rosemary extract, while rosmarinic, carnosic, and vanillic acids were found only in cookies containing rosemary extract. It was also observed that the concentration of individual polyphenolic compounds in the cookies was associated with rosemary extract content in the dough, and was significantly different (*p *< 0.05, see Table 2) between cookies baked in dry and humid air. Last but not least, the total phenolic content of the baked products was strongly correlated with their antioxidant activity.

The obtained results show a clear downward tendency in acrylamide concentration with increasing content of individual polyphenols in shortcrust cookies (as confirmed by negative correlation coefficients), which is consistent with the study by Jin et al. (2013). In experiments conducted by Bassama et al. (2010), increased concentrations of caffeic, gallic, ferulic, coumaric, and cinnamic acids did not reduce acrylamide formation in aqueous model systems containing asparagine and glucose (heated at 200 °C for 7 min). Furthermore, those authors noted a significant increase in acrylamide with the content of caffeic acid in the sample. On the other hand, Kotsiou et al. (2011) found that gallic and caffeic acids lowered the amount of acrylamide in a model system containing glucose and asparagine prepared as an emulsion. It can be therefore inferred that the reduction of acrylamide concentration by antioxidants strongly depends on the environment in which the experiments are conducted (Zhang et al. 2014). Acrylamide formation is more effectively prevented by the same phenolic acids in an emulsion system containing fat (in addition to the precursors of this carcinogenic compound). It should be noted that the carbon atoms in oxidized fats react with asparagine to form acrylamide, and so compounds inhibiting oxidation processes lower acrylamide generation (Kalita et al. 2013).

### Identification of phenolic compounds by UHPLC/DAD–ESI–MS/MS

Liquid chromatography with ESI–MS/MS was used to confirm the presence of phenolic acids in rosemary extract and in shortcrust cookies supplemented with it. Due to the fact that polyphenols contain one or more hydroxyl and/or carboxyl groups, MS data were collected in negative ionization mode.

Compound identification was based on accurate measurements of the molar masses of the quasi-molecular [M–H]^−^ ions and their fragment ions, additionally consulting the literature data (Kontogianni et al. 2013). The results obtained by ESI–MS analysis are summarized in Table 3. Caffeic and rosmarinic acids (hydroxycinnamic acids) were identified both in rosemary extract and in shortcrust cookies containing it. The presence of caffeic acid was confirmed by the deprotonated [M–H]^−^ ion with *m*/*z* 179.03 and the *m*/*z* 135.04 fragment ion resulting from the loss of carbon dioxide from the precursor ion. At a collision energy of 35 eV and an ionization voltage of 3.0 kV, rosmarinic acid underwent complete defragmentation, with the chromatogram showing only its *m*/*z* 161.02 fragment ion. Moreover, ESI–MS analysis also revealed the presence of carnosic acid (a phenolic terpene). This compound was identified on the basis of the *m*/*z* 331.19 ion [M–H]^−^ and the *m*/*z* 287.20 fragment ion resulting from the loss of carbon dioxide from the precursor ion, which then underwent further defragmentation to a daughter ion with m/z 244.15. This ion was created as a result of losing the propyl group from decarboxylated carnosic acid. Vanillic acid (a hydroxybenzoic acid) was identified on the basis of the *m*/*z* 167.03 [M–H]^−^ ion and the *m*/*z* 123.00 fragment ion (Table 3).

**Table 3:** Polyphenolic compounds found in freeze-dried aqueous rosemary extract and in shortcrust cookies made from dough supplemented with different concentrations of the extract

Retention time (min)	Name of the identified compound	[M–H]^−^ m/z	Main fragment ions m/z
2.96	Vanillic acid^a^	167.03	123.00
6.06	Caffeic acid^a^	179.03	135.04
10.14	Rosmarinic acid^a^	–	161.02
24.01	Carnosic acid^a^	331.19	287.20
			244.15

^a^Identification confirmed with the use of a standard

## Conclusion

The present study showed that both baking air humidity and the concentration of freeze-dried aqueous rosemary extract significantly reduced the amount of acrylamide formed in shortcrust cookies. At the same time, these factors enhanced the antioxidant potential of these products. Importantly, the addition of 0.1–0.5% of rosemary extract to shortcrust cookies did not significantly decrease sensory acceptability. The supplementation of shortcrust cookies with rosemary extract, which is rich in bioactive compounds, has been found advantageous due to the improved health benefits of the resulting products.
